# Widely targeted metabolomics reveals the phytoconstituent changes in *Platostoma palustre* leaves and stems at different growth stages

**DOI:** 10.3389/fpls.2024.1378881

**Published:** 2024-06-18

**Authors:** Suhua Huang, Zhining Chen, Hao Chen, Changqian Quan, Meihua Xu, Fan Wei, Danfeng Tang

**Affiliations:** ^1^ Guangxi Key Laboratory of Medicinal Resources Protection and Genetic Improvement/Guangxi Engineering Research Center of TCM Resource Intelligent Creation, National Center for TCM Inheritance and Innovation, Guangxi Botanical Garden of Medicinal Plants, Nanning, China; ^2^ College of Pharmacy, Guangxi Medical University, Nanning, China; ^3^ College of Traditional Chinese Medicine, China Pharmaceutical University, Nanjing, China; ^4^ National Engineering Research Center for Southwest Endangered Medicinal Materials Resources Development, Guangxi Botanical Garden of Medicinal Plants, Nanning, China; ^5^ College of Agriculture, Guangxi University, Nanning, China

**Keywords:** widely targeted metabolomics, *Platostoma palustre*, phytoconstituents changes, potential markers, metabolic pathways

## Abstract

*Platostoma palustre (Blume) A. J. Paton* is an important edible and medicinal plant. To gain a comprehensive and clear understanding of the variation patterns of metabolites in *P. palustre*, we employed the UPLC-MS platform along with widely targeted metabolomics techniques to analyze the metabolites in the stems and leaves of *P. palustre* at different stages. Our results revealed a total of 1228 detected metabolites, including 241 phenolic acids, 203 flavonoids, 152 lipids, 128 terpenes, 106 amino acids, 79 organic acids, 74 saccharides, 66 alkaloids, 44 lignans, etc. As the growth time increased, the differential metabolites (DAMs) mainly enriched in *P. palustre* leaves were terpenoids, phenolic acids, and lipids, while the DAMs primarily enriched in stems were terpenoids. Compared to stems, there were more differential flavonoids in leaves, and saccharides and flavonoids were significantly enriched in leaves during the S1 and S2 stages. Additionally, we identified 13, 10, and 23 potential markers in leaf, stem, and leaf vs. stem comparison groups. KEGG enrichment analysis revealed that arginine biosynthesis was the common differential metabolic pathway in different growth stages and tissues. Overall, this study comprehensively analyzed the metabolic profile information of *P. palustre*, serving as a solid foundation for its further development and utilization.

## Introduction

*Platostoma palustre (Blume) A. J. Paton*, also known as “Xiancao” or “Liangfencao” in Chinese, belongs to the Platostoma genus of Lamiaceae family and is an annual and perennial herbal plant (Tang et al., 2021). *P. palustre* is mainly distributed in Guangdong, Guangxi, Fujian, Taiwan, Zhejiang, Jiangxi, and Yunnan provinces (regions) in China, as well as in Southeast Asian countries such as Vietnam, Malaysia, India, and Indonesia (Wang and Qin, 2014; Tang et al., 2022a, 2022b). China is the world’s leading producer of *P. palustre*, a traditional medicinal and edible plant resource with high medical and nutritional value (Lin et al., 2013). Modern studies have shown that *P. palustre* possesses multiple biological activities, including antioxidation (Yen and Hung, 2000; Chen et al., 2022), antibiosis (Liu and Feng, 2008a), hypolipidemic effects (Li et al., 2010), antihypertensive effects (Yeha et al., 2009), liver injury alleviation (Hong et al., 2022), etc. Moreover, *P. palustre* has edible properties and is commonly used as bean jelly and herbal tea (Tang et al., 2023).

*P. palustre* contains various chemical components including carbohydrates, proteins, amino acids, fats, vitamins, pigments, calcium, zinc, iron, manganese, potassium, polysaccharides, phenols, triterpenoids, flavonoids, and so on (Liu and Chen, 2004). Xiancao gum is a polysaccharide with gelatinous properties found in *P. palustre* and serves as an important indicator of the quality of this Chinese herbal medicine (Lin et al., 2013). Xiancao gum is derived from the leaves, stems, and roots of *P. palustre* and is extracted using hot water or alkaline solution; Its active ingredients are similar to those found in *P. palustre*, with polysaccharide being the main constituent. Xiancao gum also contains functional components such as polyphenols, flavonoids, and terpenoids (Li J. et al., 2019). The polysaccharide content varies in different parts of *P. palustre*, with the highest content in the leaves, followed by roots, and the lowest content in the stem (Liu and Feng, 2008b). However, during the harvest period, the stem weight of the dried medicinal herbs of *P. palustre* accounts for the largest proportion of the total whole weight, followed by leaves, while the root weight is almost negligible.

Metabolomic analysis can be conducted to identify the types and quantities of metabolites based on different varieties, growth stages, tissue parts, processing methods, and compound materials (Liang et al., 2023). Metabolomics profiling is a crucial strategy for analyzing chemical components at the molecular level (Yang et al., 2021). Various modern analytical techniques, such as mass spectrometry (MS), nuclear magnetic resonance (NMR), and chromatography, are widely applied in the quantitative and qualitative evaluation of plant metabolites due to their high accuracy and sensitivity when coupled with effective chromatographic techniques that allow separation and characterization of the diversity of phytoconstituents present in medicinal plants (Li et al., 2021a). These techniques include liquid chromatography-mass spectrometry (LC-MS), gas chromatography-mass spectrometry (GC-MS), high-performance thin-layer chromatography (HPTLC), capillary electrophoresis-mass spectrometry (CE-MS), which are the most accurate techniques used for metabolites analysis and quality control of medicinal plants (Jorge et al., 2016). Currently, an ultra-performance liquid chromatography-tandem mass spectrometry (UPLC–MS/MS)-based widely targeted metabolomics analysis has been successfully used for the detection of a vast number of metabolites in many plant species, such as sweet sorghum (Zhou et al., 2020), sesame (Dossou et al., 2022), wine grape (Yu et al., 2022), *Lycium barbarum* (Wang et al., 2020) and so on. However, there has been limited research on the metabolic profiles of *P. palustre* and there are only a few reports on *P. palustre* using the LC-MS technique (Tang et al., 2021, 2023). It is worth noting that the application of UPLC–MS/MS-based widely targeted metabolomics in *P. palustre* has not been reported.

In this study, to have a comprehensive understanding of the phytoconstituents of *P. palustre*, we collected the leaves and stem segments of *P. palustre* at three growth and development stages and systematically investigated the spatiotemporal differences of metabolites in different tissues at different growth stages using a UPLC–MS/MS-based widely targeted metabolomics. The significance of this study was to reveal the metabolic characteristics and the changing law of metabolites in different growth stages and tissues of *P. palustre*, so as to provide theoretical support for the study of medicinal, edible, and ecological values of *P. palustre*, and lay the foundation for further development and utilization of *P. palustre*.

## Materials and methods

### Sample preparation

The cutting seedlings of *P. palustre* with a height of 15–20 cm were transplanted to the field on April 10, 2022, and thereafter followed normal field management. We conducted the first sampling on June 10, 2022, taking the 3^rd^-4^th^ stem segments from top to bottom and the leaves with the corresponding stem node position, while marking this position on the other branches with a rope. Subsequently, samples were taken every 2 weeks, a total of three times. We named the leaves and stem segments in chronological order as LS1, LS2, and LS3, and SS1, SS2, and SS3, respectively. All samples were immediately placed into liquid nitrogen for widely targeted metabolomic analysis, which was conducted in METWARE Biotechnology Co., Ltd (Wuhan, China).

### Sample preparation and extraction

The biological samples were freeze-dried with a vacuum freeze-dryer (Scientz-100F). The freeze-dried samples were crushed using a mixer mill (MM 400, Retsch) with a zirconia bead for 1.5 min at 30 Hz. Dissolve 50 mg of lyophilized powder with 1.2 mL 70% methanol solution, vortex 30 seconds every 30 minutes for 6 times in total. Following centrifugation at 12000 rpm for 3 min, the extracts were filtrated (SCAA-104, 0.22 μm pore size; ANPEL, Shanghai, China, http://www.anpel.com.cn/) before UPLC-MS/MS analysis.

### UPLC Conditions

The sample extracts were analyzed using a UPLC-ESI-MS/MS system (UPLC, SCIEX, ExionLC™ AD, Shanghai ABSciex Analytical Instrument Trading Co., Ltd, Shanghai, China; MS, SCIEX, Applied Biosystems 4500 Q TRAP, Shanghai ABSciex Analytical Instrument Trading Co., Ltd, Shanghai, China). The analytical conditions were as follows, UPLC: column, Agilent SB-C18 (1.8 µm, 2.1 mm * 100 mm); The mobile phase consisted of solvent A, pure water with 0.1% formic acid, and solvent B, acetonitrile with 0.1% formic acid. Sample measurements were performed with a gradient program that employed the starting conditions of 95% A, 5% B. Within 9 min, a linear gradient to 5% A, 95% B was programmed, and a composition of 5% A, 95% B was kept for 1 min. Subsequently, a composition of 95% A and 5.0% B was adjusted within 1.1 min and kept for 2.9 min. The flow velocity was set as 0.35 mL per minute; The column oven was set to 40°C; The injection volume was 4 μL. The effluent was alternatively connected to an ESI-triple quadrupole-linear ion trap (QTRAP)-MS.

### ESI-Q TRAP-MS/MS

The ESI source operation parameters were as follows: source temperature 550°C; ion spray voltage (IS) 5500 V (positive ion mode)/-4500 V (negative ion mode); ion source gas I (GSI), gas II (GSII), curtain gas (CUR) were set at 50, 60, and 25 psi, respectively; the collision-activated dissociation (CAD) was high. QQQ scans were acquired as MRM experiments with collision gas (nitrogen) set to medium. DP (declustering potential) and CE (collision energy) for individual MRM transitions were done with further DP and CE optimization. A specific set of MRM transitions was monitored for each period according to the metabolites eluted within this period.

### Principal component analysis

Principal component analysis (PCA) was performed using the built-in statistical function prcomp of the R software (https://www.r-project.org/), and the parameter of the prcomp function was set to scale=True, indicating that the data were subjected to UV (unit variance scaling).

### Hierarchical cluster analysis and Pearson correlation coefficients

The hierarchical cluster analysis (HCA) results of samples and metabolites were presented as heatmaps with dendrograms, while the Pearson correlation coefficients (PCC) between samples were calculated by the cor function in R and presented as only heatmaps. Both HCA and PCC were carried out by R package ComplexHeatmap. For HCA, normalized signal intensities of metabolites (unit variance scaling) are visualized as a color spectrum.

### Differential metabolites selection

For two-group analysis, differential metabolites were determined by Variable importance in projection (VIP) ≥ 1 and absolute |Log_2_Fold change (FC)| ≥ 1.0. VIP values were extracted from the OPLS-DA (OrthogonalPartialLeast Squares-DiscriminantAnalysis) results, which also contained score plots and permutation plots, and were generated using the R package MetaboAnalystR. The data was log transform (Log_2_) and mean centering before OPLS-DA. In order to avoid overfitting, a permutation test (200 permutations) was conducted.

### KEGG annotation and enrichment analysis

The identified metabolites were annotated using the KEGG Compound database (http://www.kegg.jp/kegg/compound/), and the annotated metabolites were then mapped to the KEGG Pathway database (http://www.kegg.jp/kegg/pathway.html). Pathways with significantly regulated metabolites mapped were then fed into MSEA (metabolite sets enrichment analysis), their significance was determined by hypergeometric test^s^ p-values.

### Data analysis

In all the analysis contents of metabolomics in this study, UV (unit variance scaling) and Zero-centered (Ctr) were taken to process the data during the analysis. In addition, GraphPad Prism 7 and WPS software were employed for data processing and graph analysis.

## Results

### Qualitative and quantitative analysis of metabolites in *P. palustre*

**Figure 1:**
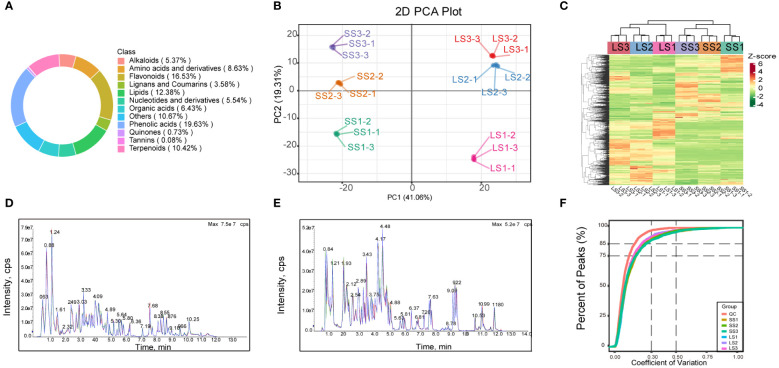
The metabolite class composition, PCA plot, clustering heat-map, TIC plots, and CV value distribution map in this study. **(A)** The circular plot of overall metabolite class composition, with values in parentheses indicating the percentage of each categorical metabolite to the total metabolite; **(B)** The PCA plot for each group of samples. **(C)** The cluster heatmap of the overall samples of each group. **(D, E)** The total ions current (TIC) plots of mixed-sample QC samples in positive and negative ion modes, respectively, with the horizontal coordinate being the retention time (Rt) of the metabolite assay, and the vertical coordinate being the intensity of the ion current of the ion assay (intensity in cps, count per second). **(F)** The CV value distribution map for all samples, with the horizontal axis representing the CV value and the vertical axis representing the proportion of the number of substances less than the corresponding CV value to the total number of substances. Different colors represented different grouped samples, and QC represented quality control samples.

In this study, to obtain a comprehensive and clear understanding of the variation patterns of metabolites in *P. palustre*, we employed the UPLC-MS platform together with widely targeted metabolomics techniques to identify and analyze the metabolites in the stems and leaves of *P. palustre* at different growth stages. The results showed that a total of 1228 metabolites were detected, including 241 phenolic acids, 203 flavonoids, 152 lipids, 128 terpenes, 66 alkaloids, 79 organic acids, 44 lignans, 9 quinones, 106 amino acids, 68 nucleotide derivatives, 74 saccharides, 1 tannin and so on (**Figure 1A**). Among these, 1220, 1220, and 1216 metabolites were detected in the stems at three growth stages (SS1, SS2, SS3), while 1224, 1220, and 1225 metabolites were identified in the leaves at three growth stages (LS1, LS2, LS3), respectively. These results indicated that the types and quantities of metabolites in the stems and leaves of *P. palustre* at different growth stages were generally similar (**Supplementary Table 1**).

### Principal component analysis of all samples

The PCA results in this study revealed samples from the same group clustering together, with a distinct separation trend observed between samples from different groups. These findings indicated high reproducibility of the samples and significant differences in metabolite features across groups. The PCA model analysis yielded a total explanation rate of 60.37% for the samples (**Figure 1B**). Moreover, a clustering heat map can display the expression abundance of metabolites in *P. palustre* (**Figure 1C**). The clustering analysis revealed substantial differences in metabolites among groups, which could be categorized into four clusters: one cluster consisted of metabolite profiles from *P. palustre* leaves, where LS1 formed a distinct cluster and LS2 and LS3 formed another cluster; another cluster comprised metabolite profiles from *P. palustre* stems, with SS1 forming a separate cluster and SS2 and SS3 forming the other cluster. The three biological replicates within each treatment group also exhibited clustering, suggesting the high homogeneity of the data in this study. The overlapping display analysis of the total ion flow chart from different quality control samples demonstrated the high stability of the instrument and the reliability of the data results, which can be utilized for subsequent analysis (**Figures 1D, E**). Based on the coefficient of variation (CV) as an indicator of data dispersion, the results demonstrated that over 75% of substances in the QC samples had a CV value below 0.3, suggesting the stability and reliability of the experimental data (**Figure 1F**).

### OPLS-DA analysis and permutation test analysis

**Figure 2:**
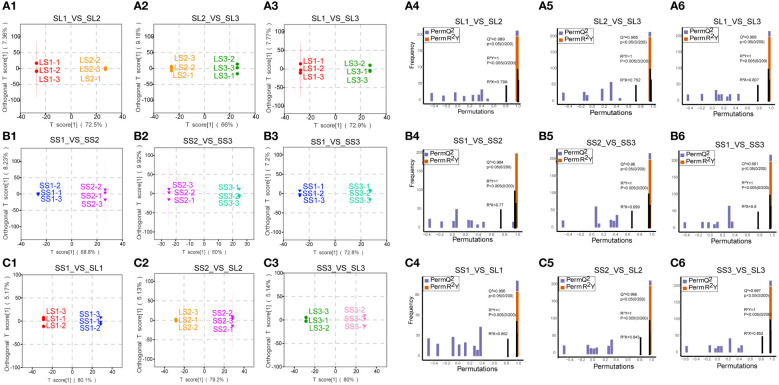
The OPLS-DA score plot and OPLS-DA model validation. **(A1–A3)** The OPLS-DA score plot of SL1 vs. SL2, SL2 vs. SL3, SL1 vs. SL3. **(B1–B3)** The OPLS-DA score plot of SS1 vs. SS2, SS2 vs. SS3, SS1 vs. SS3. **(C1–C3)** The OPLS-DA score plot of SS1 vs. SL1, SS2 vs. SL2, SS3 vs. SL3. **(A4–A6)** The OPLS-DA model validation of SL1 vs. SL2, SL2 vs. SL3, SL1 vs. SL3. **(B4–B6)** The OPLS-DA model validation of SS1 vs. SS2, SS2 vs. SS3, SS1 vs. SS3. **(C4–C6)** The OPLS-DA model validation of SS1 vs. SL1, SS2 vs. SL2, SS3 vs. SL3. The horizontal axis represented the R^2^Y and Q^2^ values of the model, while the vertical axis represented the frequency of model classification effects in 200 randomly arranged combination experiments. In the figure, orange represented the random grouping model R^2^Y, purple represented the random grouping model Q^2^, and the black arrow represented the R^2^X, R^2^Y, and Q^2^ values of the original model.

We divided the samples into nine groups: SS1 vs. SS2, SS1 vs. SS3, SS2 vs. SS3, LS1 vs. LS2, LS1 vs. LS3, LS2 vs. LS3, SS1 vs. LS1, SS2 vs. LS2, and SS3 vs. LS3. The OPLS-DA model was subjected to 200 random permutation experiments, and both Q^2^ and R^2^Y were greater than 0.9, indicating the stability and reliability of the model. The OPLS-DA score plot depicted a distinct separation of *P. palustre* samples, indicating significant differences in metabolic features across different groups (**Figure 2**).

### Identification of differential metabolites

**Figure 3:**
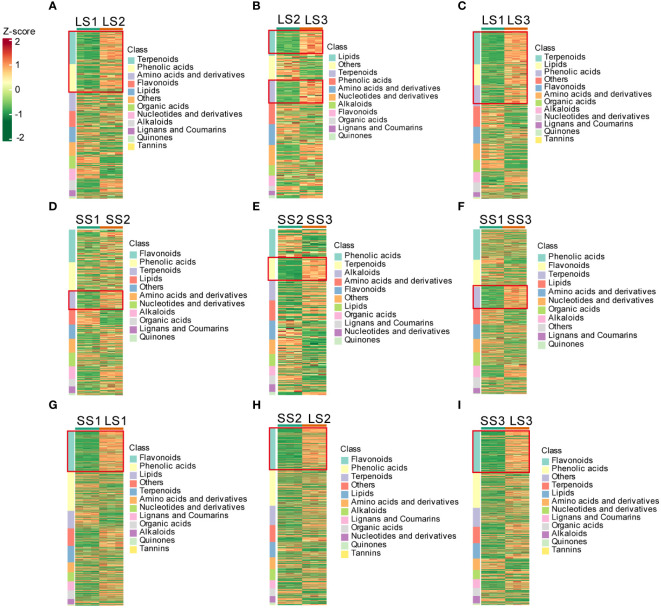
Cluster heatmaps of differential metabolites in different tissues at different growth stages. **(A–C)** Cluster heatmaps of differential metabolites in leaves between different comparison groups. **(D–F)** Cluster heatmaps of differential metabolites in stems between different comparison groups. **(G–I)** Cluster heatmaps of differential metabolites between leaves and stems at the same growth stages.

Variable importance in projection (VIP) and fold change (FC) were combined to further screen out differential metabolites. The metabolites that met both VIP ≥ 1, as well as FC ≥ 2 or FC ≤ 0.5, were considered differential metabolites, and the metabolite set characteristics of *P. palustre* among different groups were analyzed (**Figure 3**; **Supplementary Table 2**).

During different growth periods of *P. palustre*, the main differential metabolites in leaf tissues were terpenoids, phenolic acids, and lipids (**Figures 3A–C**). The LS1 vs. LS2 comparison group contained a total of 418 differential metabolites, with the largest differences observed in terpenoids (82 terpenoids: 11 accumulated in LS1 and 71 accumulated in LS2) and phenolic acids (70 phenolic acids: 19 accumulated in LS1 and 51 accumulated in LS2). The LS2 vs. LS3 comparison group consisted of 274 differential metabolites, with the largest differences observed in terpenes (38 terpenoids: 3 accumulated in LS2 and 35 accumulated in LS3) and lipids (41 lipids: 2 accumulated in LS2 and 39 accumulated in LS3). The LS1 vs. LS3 comparison group revealed a total of 441 differential metabolites, with the largest differences observed in terpenoids (88 terpenoids: 5 accumulated in LS1 and 83 accumulated in LS3), lipids (55 lipids: 10 accumulated in LS1 and 45 accumulated in LS3), and phenolic acids (55 phenolic acids: 14 accumulated in LS1 and 41 accumulated in LS3) (**Supplementary Table 2**).

Terpenoids and lipids were the primary differential metabolites in the stem segments during different growth stages of *P. palustre* (**Figures 3D–F**), and their accumulation significantly increased with the prolongation of the growth time. The SS1 vs. SS2 comparison group identified 40 terpenoids and 39 lipids among the 396 differential metabolites, with 7 terpenoids and 4 lipids accumulated in SS1, and 33 terpenoids and 35 lipids accumulated in SS2, respectively. The SS2 vs. SS3 comparison group revealed 36 terpenoids and 45 phenolic acids among the 263 differential metabolites, with 2 terpenoids and 31 phenolic acids accumulated in SS2, and 34 terpenoids and 14 phenolic acids accumulated in SS3, respectively. In the SS1 vs. SS3 comparison group, 63 terpenoids out of 482 differential metabolites were detected, with 7 terpenoids accumulated in SS1, and 56 terpenoids accumulated in SS3, respectively (**Supplementary Table 2**).

The SS1 vs. LS1, SS2 vs. LS2, and SS3 vs. LS3 comparison groups contained 658, 612, and 643 differential metabolites, respectively. Flavonoids were the most abundant and exhibited the most pronounced trend of change among these metabolites, with 152, 147, and 155 flavonoids accounting for 23.10%, 24.02%, and 24.11% of the total differential metabolites in the SS1 vs. LS1, SS2 vs. LS2, and SS3 vs. LS3 comparison groups, respectively (**Figures 3G–I**; **Supplementary Table 2**).

### Metabolic characteristics of different tissues and growth stages of *P. palustre*

**Figure 4:**
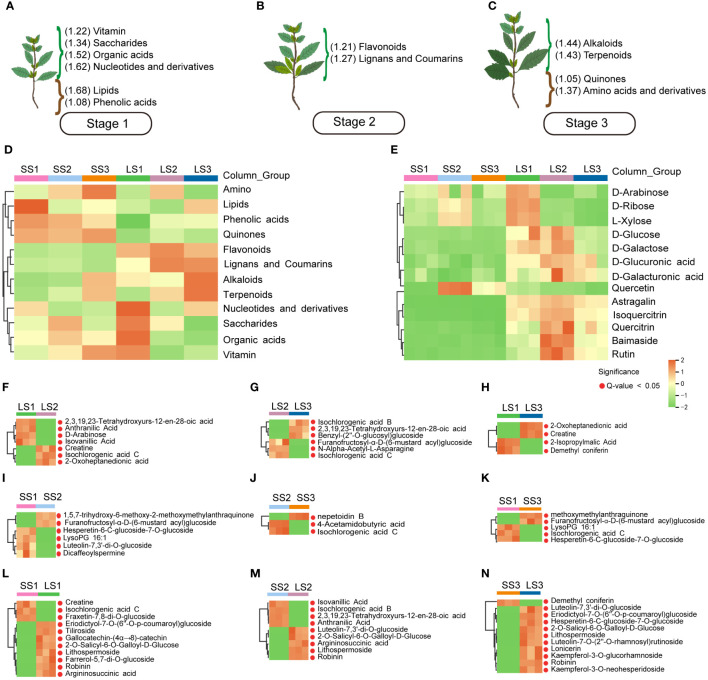
The main metabolites and heatmaps of potential markers in different tissues and growth stages of *P. palustre*. **(A–C)** The enrichment of different kinds of metabolites at different growth stages of *P. palustre* (The values in parentheses represent the standardized relative content of metabolites, and the magnitude of the values represents the relative content); **(D)** The distribution heatmap of various metabolites in different tissues and growth stages of *P. palustre*; **(E)** The cluster heatmaps of the main monosaccharides and flavonoids in different tissues and growth stages of *P. palustre*. **(F–N)** The cluster heatmaps of the potential biomarkers in different comparison groups.

To visually illustrate the enrichment characteristics of various metabolites in *P. palustre*, we displayed the metabolites that showed the most pronounced enrichment trends in the three growth stages. In the S1 stage, the leaves exhibited higher enrichment of organic acids, nucleotides and their derivatives, and saccharides, while the stem segments showed higher enrichment of lipids and phenolic acids (**Figure 4A**). In the S2 stage, leaves showed high levels of enrichment in flavonoids, lignans, and coumarins (**Figure 4B**). In the S3 stage, leaves exhibited a higher enrichment of alkaloids and terpenoids, while stem segments showed a higher enrichment of quinones, amino acids and their derivatives (**Figure 4C**). The clustered heat map of the total metabolite content in each sample was shown in **Figure 4D**. It is worth mentioning that flavonoids showed significant enrichment in leaf tissues throughout the growth stages of *P. palustre*, compared to stem segments.

In addition, polysaccharides (composed of different monosaccharides) and flavonoids were the primary chemical components of *P. palustre*, and they directly influenced its quality and efficacy. We further analyzed the metabolic characteristics of these two types of compounds in different tissues and growth stages of *P. palustre*. The results indicated that monosaccharides were primarily enriched in the leaf tissue of *P. palustre*. Among them, the D-arabinose, D-ribose, and L-xylose were predominantly enriched in the leaf tissue during the S1 stage. In the leaf tissue during the S2 stage, there was a high enrichment of D-glucose, D-galactose, D-glucuronic acid, and D-galactonic acid. Additionally, D-arabinose, D-ribose, and L-xylose exhibited a certain level of enrichment in the stem tissue during the S2 stage (**Figure 4E**). Flavonoids were primarily enriched in the leaf tissue compared to the stem tissue, with astragalin, isoquercitrin, quercitrin, baimaside, and rutin exhibiting the most significant enrichment trend during the S2 stage. In contrast, quercetin showed high enrichment in the stem tissue during the S2 stage (**Figure 4E**).

### Screening for potential markers

**Figure 5:**
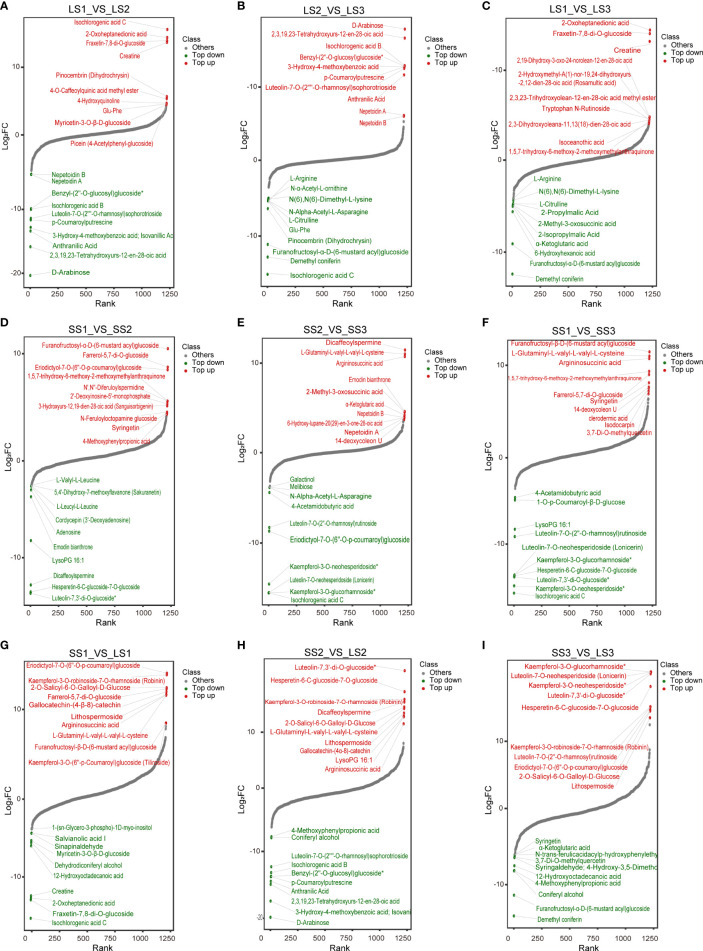
Dynamic distribution of metabolite content differences in different comparison groups. **(A–C)** Dynamic distribution map of differential metabolites in LS1 _ VS _ LS2, LS2 _ VS _ LS3, and LS1 _ VS _ LS3 comparison groups, respectively. **(D–F)** Dynamic distribution map of differential metabolites in SS1 _ VS _ SS2, SS2 _ VS _ SS3, and SS1 _ VS _ SS3 comparison groups, respectively. **(G–I)** Dynamic distribution map of differential metabolites in LS1 _ VS _ SS1, LS2 _ VS _ SS2, and LS3 _ VS _ SS3 comparison groups, respectively. The horizontal coordinate of the graph represented the cumulative number of substances in order of FC from smallest to largest, and the vertical coordinate represented the logarithmic value of FC with 2 as the base, and each point represented a substance. Green points represented the top 10 substances in down-regulation, and the red points represented the top 10 substances in up-regulation.

**Figure 6:**
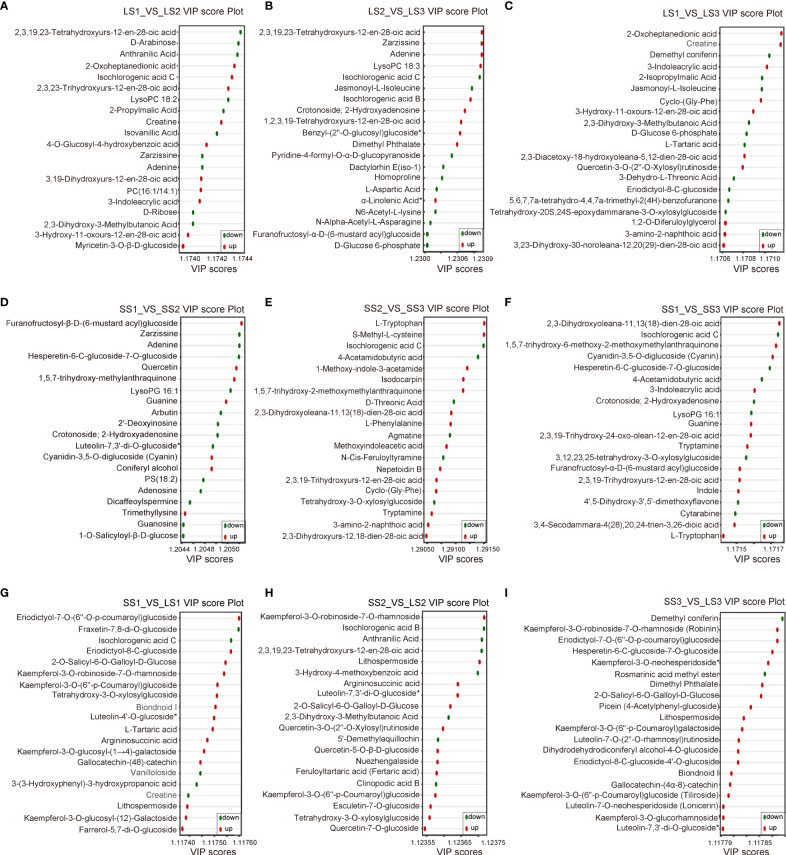
The VIP score plots of differential metabolites in different comparison groups. **(A–C)** VIP score plots of differential metabolites in LS1 _ VS _ LS2, LS2 _ VS _ LS3, and LS1 _ VS _ LS3 comparison groups, respectively. **(D–F)** VIP score plots of differential metabolites in SS1 _ VS _ SS2, SS2 _ VS _ SS3, and SS1 _ VS _ SS3 comparison groups, respectively. **(G–I)** VIP score plots of differential metabolites in LS1 _ VS _ SS1, LS2 _ VS _ SS2, and LS3 _ VS _ SS3 comparison groups, respectively. The horizontal coordinate represented the VIP value, and the vertical coordinate represented the differential metabolites. Red dots represented the up-regulated differential metabolites, green dots represented the down-regulated differential metabolites, and yellow dots represented the metabolites that had significant differences in three or more different comparison groups.

To identify the metabolites that contributed the most to the differences between the comparison groups, we considered the metabolites with the top 10 positive and negative Log_2_ FC values (**Figure 5**) and the top 20 VIP values (**Figure 6**) as potential markers. These potential markers were identified based on the nine comparison groups (**Figures 4F–N**; **Supplementary Table 3**).

In the LS1 vs. LS2 group, 7 potential markers were found, including Isochlorogenic acid C (Phenolic acids, Log_2_ FC=15.6), 2-Oxoheptanedionic acid (Organic acids, Log_2_ FC=14.42), Creatine (Organic acids, Log_2_ FC=13.7), D-Arabinose (Saccharides, Log_2_ FC=-20.71), 2,3,19,23-Tetrahydroxyurs-12-en-28-oic acid (Terpenoids, Log_2_ FC=-16.46), Isovanillic acid (Phenolic acids, Log_2_ FC=-13.61), and Anthranilic acid (Phenolic acids, Log_2_ FC=-14.14) (**Figure 4F**). In the LS2 vs. LS3 group, 6 potential markers were identified, including 2,3,19,23-Tetrahydroxyurs-12-en-28-oic acid (Terpenoids, Log_2_ FC=16.74), Benzyl-(2’’-O-glucosyl) glucoside (Phenolic acids, Log_2_ FC=12.88), Isochlorogenic acid B (Phenolic acids, Log_2_ FC=12.97), Isochlorogenic acid C (Phenolic acids, Log_2_ FC=-15.6), Furanofructosyl-α-D-(6-mustard acyl) glucoside (Phenolic acids, Log_2_ FC=-11.51), and N-Alpha-Acetyl-L-Asparagine (Phenolic acids, Log_2_ FC=-5.51) (**Figure 4G**). 6 potential markers were found in LS1 vs. LS3, such as Creatine (Organic acids, Log_2_ FC=13.47), 2-Oxoheptanedionic acid (Organic acids, Log_2_ FC=14.73), Demethyl coniferin (Phenolic acids, Log_2_ FC=-12.32), and 2-Isopropylmalic Acid (Organic acids, Log_2_ FC=-4.69) (**Figure 4H**).

There were 6, 3, and 5 potential markers in SS1 vs. SS2 [Furanofructosyl-α-D-(6-mustard acyl) glucoside (Phenolic acids, Log_2_ FC=10.78), Dicaffeoylspermine (Alkaloids, Log_2_ FC=-12.72), Luteolin-7,3’-di-O-glucoside (Flavonoids, Log_2_ FC=-13.55), Hesperetin-6-C-glucoside-7-O-glucoside (Flavonoids, Log_2_ FC=-13.41), 1,5,7-trihydroxy-6-methoxy-2-methoxymethylanthraquinone (Anthraquinone, Log_2_ FC=5.57), and LysoPG 16:1 (Lipids, Log_2_ FC=-8.31)], SS2 vs. SS3 [Nepetoidin B (Phenolic acids, Log_2_ FC=3.74), Isochlorogenic acid C (Phenolic acids, Log_2_ FC=-15.43), and 4-Acetamidobutyric acid (Organic acids, Log_2_ FC=-4.46)], and SS1 vs. SS3 [Furanofructosyl-α-D-(6-mustard acyl) glucoside (Phenolic acids, Log_2_ FC=11.15), Isochlorogenic acid C (Phenolic acids, Log_2_ FC=-15.32), Hesperetin-6-C-glucoside-7-O-glucoside (Flavonoids, Log_2_ FC=-13.41), 1,5,7-trihydroxy-6-methoxy-2-methoxymethylanthraquinone (Anthraquinone, Log_2_ FC=9.01), and LysoPG 16:1 (Lipids, Log_2_ FC=-8.31)], respectively (**Figures 4I–K**).

In SS1 vs. LS1, a total of 11 potential markers, including Eriodictyol-7-O-(6’’-O-p-coumaroyl) glucoside (Flavonoids, Log_2_ FC=15.18), Gallocatechin-(4α→8)-catechin (Flavonoids, Log_2_ FC=12.88), Kaempferol-3-O-robinoside-7-O-rhamnoside (Robinin) (Flavonoids, Log_2_ FC=14.97), Kaempferol-3-O-(6’’-p-Coumaroyl) glucoside (Tiliroside) (Flavonoids, Log_2_ FC=8.95), Farrerol-5,7-di-O-glucoside (Flavonoids, Log_2_ FC=13.23), Lithospermoside (Alkaloids, Log_2_ FC=12.52), 2-O-Salicyl-6-O-Galloyl-D-Glucose (Phenolic acids, Log_2_ FC=13.37), Argininosuccinic acid (Organic acids, Log_2_ FC=12.48), Creatine (Organic acids, Log_2_ FC=-12.53), Fraxetin-7,8-di-O-glucoside (Lignans and Coumarins, Log_2_ FC=-13.02), and Isochlorogenic acid C (Phenolic acids, Log_2_ FC=-15.32) were identified (**Figure 4L**). In SS2 vs. LS2, there were 9 potential markers, like Luteolin-7,3’-di-O-glucoside (Flavonoids, Log_2_ FC=19.85), Kaempferol-3-O-robinoside-7-O-rhamnoside (Robinin) (Flavonoids, Log_2_ FC=15.24), Lithospermoside (Alkaloids, Log_2_ FC=13.02), Argininosuccinic acid (Organic acids, Log_2_ FC=11.3), 2-O-Salicyl-6-O-Galloyl-D-Glucose (Phenolic acids, Log_2_ FC=14.01), Isochlorogenic acid B (Phenolic acids, Log_2_ FC=-12.65), 3-Hydroxy-4-methoxybenzoic acid (Isovanillic Acid) (Phenolic acids, Log_2_ FC=-17.23), Anthranilic Acid (Phenolic acids, Log_2_ FC=-14.05), and 2,3,19,23-Tetrahydroxyurs-12-en-28-oic acid (Terpenoids, Log2 FC=-14.55) (**Figure 4M**). Moreover, in SS3 vs. LS3, 11 potential markers were found, containing Eriodictyol-7-O-(6’’-O-p-coumaroyl) glucoside (Flavonoids, Log_2_ FC=14.44), Luteolin-7,3’-di-O-glucoside (Flavonoids, Log_2_ FC=19.45), Luteolin-7-O-neohesperidoside (Lonicerin) (Flavonoids, Log_2_ FC=19.69), Kaempferol-3-O-glucorhamnoside (Flavonoids, Log_2_ FC=19.69), Luteolin-7-O-(2’’-O-rhamnosyl) rutinoside (Flavonoids, Log_2_ FC=14.82), Kaempferol-3-O-robinoside-7-O-rhamnoside (Robinin) (Flavonoids, Log_2_ FC=14.9), Hesperetin-6-C-glucoside-7-O-glucoside (Flavonoids, Log_2_ FC=17.69), Kaempferol-3-O-neohesperidoside (Flavonoids, Log_2_ FC=19.68), Lithospermoside (Alkaloids, Log_2_ FC=13.37), 2-O-Salicyl-6-O-Galloyl-D-Glucose (Phenolic acids, Log_2_ FC=14.3), and Demethyl coniferin (Phenolic acids, Log_2_ FC=-14.02) (**Figure 4N**).

### KEGG enrichment analysis

**Figure 7:**
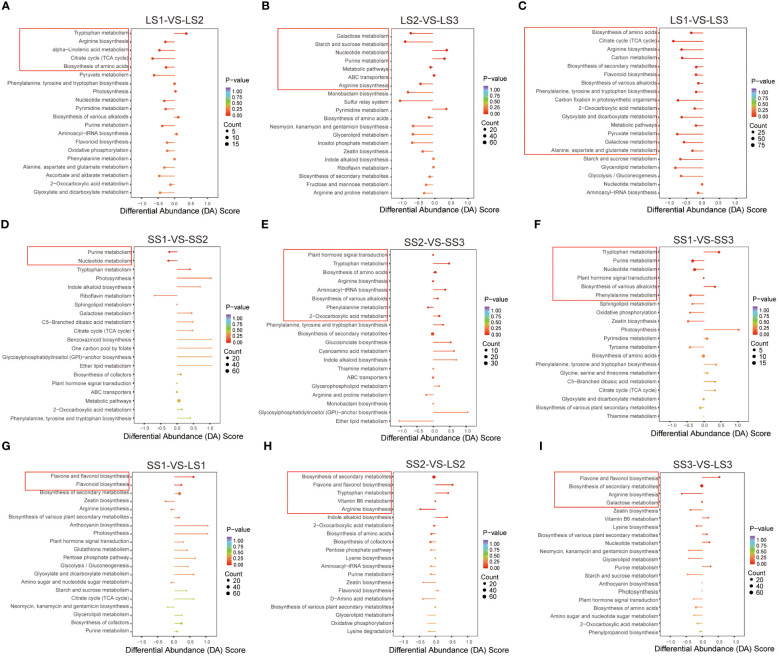
KEGG enrichment analysis in different comparison groups. **(A–C)** KEGG enrichment analysis of differential metabolites in LS1 _ VS _ LS2, LS2 _ VS _ LS3, and LS1 _ VS _ LS3 comparison groups, respectively. **(D–F)** KEGG enrichment analysis of differential metabolites in SS1 _ VS _ SS2, SS2 _ VS _ SS3, and SS1 _ VS _ SS3 comparison groups, respectively. **(G–I)** KEGG enrichment analysis of differential metabolites in LS1 _ VS _ SS1, LS2 _ VS _ SS2, and LS3 _ VS _ SS3 comparison groups, respectively. DA Score value greater than zero indicated that the metabolites in the pathway were generally up-regulated, and less than zero indicated that the metabolites in the pathway were generally down-regulated. The dots at the end of the line segment represented the number of metabolites.

In this study, we identified the pathways with *p* < 0.05 as significantly enriched. We analyzed the overall changes in differential metabolites within the pathways using the differential abundance score (DA Score). In the LS1 vs. LS2 comparison, we observed five significantly enriched pathways: up-regulation of the tryptophan metabolism pathway, down-regulation of arginine biosynthesis, alpha-linolenic acid metabolism, citrate cycle (TCA cycle), and biosynthesis of amino acids pathways (**Figure 7A**). In the LS2 vs. LS3 comparison, we observed seven significantly enriched pathways: up-regulation of nucleotide metabolism and purine metabolism, down-regulation of galactose metabolism, starch and sucrose metabolism, and arginine biosynthesis. The metabolic pathways and ABC transporters showed a similar trend in differential abundance (**Figure 7B**). As expected, we identified 15 significantly enriched pathways in the LS1 vs. LS3 comparison, with a significant down-regulation in differential abundance. These pathways mainly included biosynthesis of amino acids, flavonoid biosynthesis, carbon metabolism, and others (**Figure 7C**).

The SS1 vs. SS2, SS2 vs. SS3, and SS1 vs. SS3 comparison groups exhibited 2, 8, and 6 significantly enriched pathways, respectively (**Figures 7D–F**). In the SS1 vs. SS2 comparison group, purine metabolism and nucleotide metabolism were down-regulated, while tryptophan metabolism, biosynthesis of amino acids, biosynthesis of various alkaloids, aminoacyl-tRNA biosynthesis, and 2-oxocarboxylic acid metabolism were up-regulated. In the SS2 vs. SS3 comparison group, phenylalanine metabolism was down-regulated, showing similar trends in differential abundance as arginine biosynthesis and plant hormone signal transduction. Furthermore, in the SS1 vs. SS3 comparison group, tryptophan metabolism and biosynthesis of various alkaloids were up-regulated, whereas purine metabolism, nucleotide metabolism, and phenylalanine metabolism were down-regulated.

In the SS1 vs. LS1 comparison group, there were two significantly enriched pathways: flavone and flavonol biosynthesis and flavonoid biosynthesis showed up-regulation. In the SS2 vs. LS2 comparison group, there were five significantly enriched pathways: flavone and flavonol biosynthesis and tryptophan metabolism showed up-regulation, while arginine biosynthesis showed down-regulation. Biosynthesis of secondary metabolites and Vitamin B6 metabolism showed no change. In the SS3 vs. LS3 comparison group, there were four significantly enriched pathways: flavone and flavonol biosynthesis showed up-regulation, while arginine biosynthesis showed down-regulation. Biosynthesis of secondary metabolites and Galactose metabolism showed no change (**Figures 7G–I**).

### The metabolic map of the key metabolic pathways in this study

**Figure 8:**
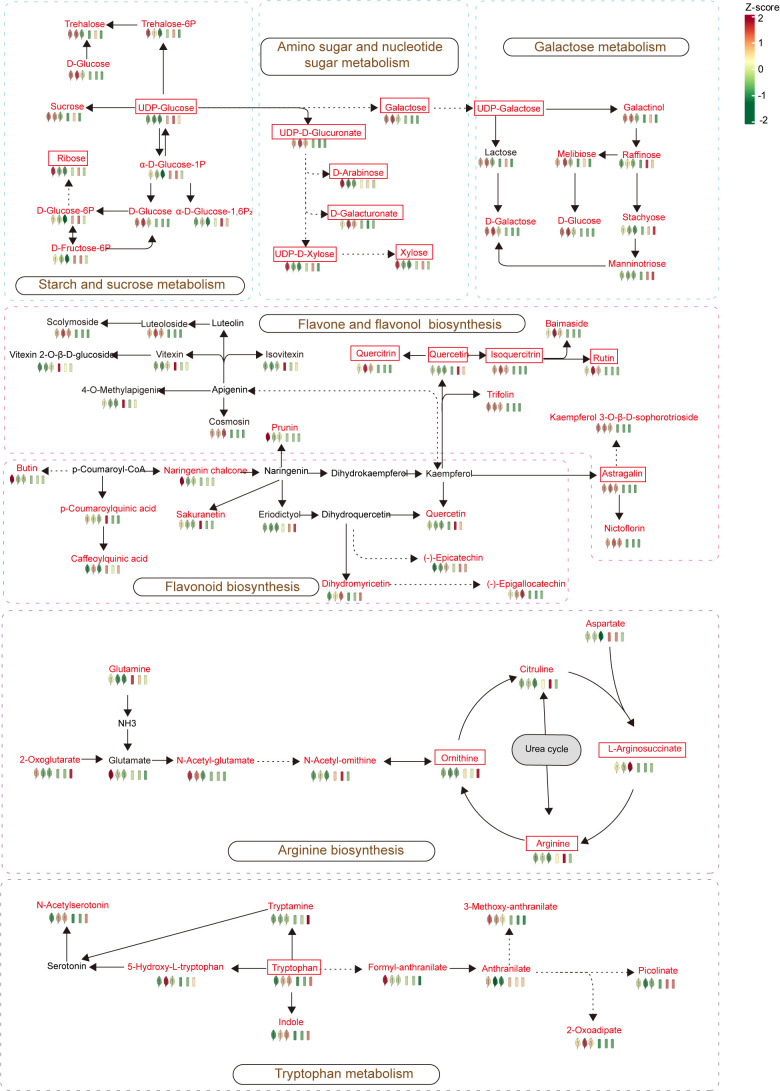
Metabolic maps of the important metabolic pathways in this study. The red font represents differential metabolites, while the framed font represents monosaccharides and important flavonoids in *P. palustre*, as well as important metabolites in the arginine and tryptophan metabolic pathways.

According to the KEGG enrichment analysis results, arginine biosynthesis was a common differential pathway between leaf comparison groups at different stages, while tryptophan metabolism was a common differential pathway between stem comparison groups at different stages. In addition, tryptophan metabolism and arginine biosynthesis were metabolic pathways with significant differences between the stem-leaf comparison groups, and flavone and flavonol biosynthesis was also the most significant common differential pathway between the stem vs. leaf comparison groups. Therefore, these three metabolic pathways (arginine biosynthesis, tryptophan metabolism, and flavone and flavonol biosynthesis) were the representative metabolic pathways in the growth and development of *P. palustre*, and were of great significance for the overall growth and development of *P. palustre*. It was especially worth mentioning that polysaccharides and flavonoids were the main chemical constituents of *P. palustre*, which directly affected the quality and efficacy of *P. palustre*, so the analysis of the synthesis pathways of polysaccharides and flavonoids was of great value. Overall, based on the KEGG database information, a metabolic pathway map of seven crucial pathways, including tryptophan metabolism, arginine biosynthesis, flavone and flavonol biosynthesis, flavonoid biosynthesis, starch and sucrose metabolism, amino sugar and nucleotide sugar metabolism, galactose metabolism in different tissues and growth stages of *P. palustre* was established in this study (**Figure 8**).

## Discussion

### The technological advantage of widely targeted metabolomics

*Platostoma palustre (Blume) A. J. Paton* is an important edible and medicinal plant in China, as well as in Southeast Asian countries such as Vietnam, Malaysia, India, and Indonesia (Wang and Qin, 2014; Tang et al., 2022a, 2022b). As previously mentioned, metabolomics profiling is an important strategy for analyzing the chemical components of medicinal plants at the molecular level (Yang et al., 2021) and it can be used to identify the types and quantities of metabolites based on different varieties, growth stages, tissue parts, processing methods, and compound materials (Liang et al., 2023). Widely targeted metabolomics has been widely recognized for accurately characterizing metabolites with high throughput, high sensitivity, and broad coverage (Sawada et al., 2009). As an emerging technology combining both non-targeted and targeted metabolomics techniques, widely targeted metabolomics has the advantages of broader coverage and higher sensitivity compared with traditional metabolomics techniques (Chen et al., 2013). Using non-targeted metabolomics, a total of 174 (Tang et al., 2023), 184 (Tang et al., 2021), and 177 (Chen et al., 2024) metabolites were identified in different treatments in *P. palustre*. However, in this study, we identified 1228 metabolites using widely targeted metabolomics. This indicated that more accurate metabolite information was captured by widely targeted metabolomics. As a result, this technique is more conducive to the analysis of chemical composition in medicinal plants, although it is currently a bit expensive.

### Distribution and enrichment characteristics of polysaccharides in *P. palustre*

Most medicinal plants are rich in polysaccharides, and their pharmacological properties can be summarized as immunomodulatory, antitumor, anti-inflammatory, antihypertensive and antihyperlipidemic, antioxidant, and antimicrobial properties, which have been shown to have the effects of enhancing the immune system, preventing cancer, inflammation, and infection (Taoerdahong et al., 2023). *P. palustre* polysaccharides have a variety of functional activities, including antioxidant, regulation of intestinal flora, hypoglycemic, hypolipidemic, hepatoprotective, immunomodulatory, and so on (Tang et al., 2023). In addition, the polysaccharides are also the main components of the gum of *P. palustre*, which is an important indicator of the quality of *P. palustre* (Lin et al., 2013). *P. palustre* polysaccharides consist of eight monosaccharides, including galacturonic acid, glucose, galactose, xylose, mannose, rhamnose, ribose, and glucuronic acid, with molar percentages of 28.4, 26.5, 16.4, 10.6, 7.4, 5.7, 4.2, and 0.9%, respectively (Lin et al., 2013; Zhang et al., 2013). In this study, the results showed that the monosaccharide compounds were mainly enriched in the leaf tissues of *P. palustre* (D-arabinose, D-ribose, and L-xylose were highly enriched at the S1 stage, and D-glucose, D-galactose, D-glucoronic acid, and D-galacturonic acid were highly enriched at the S2 stage) (**Figure 4E**). Among them, D-glucose, D-galactose, D-glucoronic acid, and D-galacturonic acid had the highest percentage of content, accounting for about 80% of the total polysaccharide content. These four monosaccharides accumulated the highest in LS2, therefore, when considering polysaccharides as a harvesting index, the S2 stage might be an optimal harvesting time. In addition, analysis of the metabolic pathways involved in monosaccharide synthesis in *P. palustre* revealed that the pathways involved in monosaccharide biosynthesis and metabolism were mainly starch and sucrose metabolism, amino sugar and nucleotide sugar metabolism, and galactose metabolism pathways. It was indicated that these metabolic pathways might directly affect the synthesis and metabolism of polysaccharides in *P. palustre*, leading to the differential enrichment of polysaccharides in different tissues and the growth and development stage of *P. palustre*.

### Distribution and enrichment characteristics of flavonoids in *P. palustre*

Flavonoids are distributed in almost all plant tissues and have many pharmacological activities, especially quercetin, which has strong pharmacological activity in preventing DNA damage caused by various cancer factors (George et al., 2017). In addition, flavonoids have a variety of biological functions and play an important role in regulating plant growth and development, resisting various stresses, and regulating plant flower color (Wang et al., 2021b; Zhuang et al., 2023). In our study, during the growth process of *P. palustre*, flavonoids were enriched in both stems and leaves with the extension of time, among which the leaves had the most obvious enrichment of flavonoids. It was worth mentioning that in this study, Quercetin was significantly enriched in stem tissues at the S2 stage. This suggested that the stems of *P. palustre* might also have medicinal value in preventing DNA damage. Overall, the leaves of *P. palustre* might have higher medicinal value, a conclusion that was consistent with the findings of other research (Liu and Feng, 2008b) (**Figure 4E**). If flavonoid compounds were used as a harvesting indicator, *P. palustre* harbored the most variety and higher content of flavonoids at the S2 stage, which was the optimal harvesting time. This stage was consistent with the period when considering polysaccharides as a harvesting index. Therefore, we suggested that this period (S2) be designated as the optimal harvesting period, which would optimize the utilization of *P. palustre*. In addition, analysis of the synthesis pathway of flavonoids in *P. palustre* revealed that the pathways of flavone and flavonol biosynthesis and flavonoid biosynthesis were highly up-regulated in the leaf tissues, which might be the main reason for the differences in flavonoids between the leaf tissues and stem tissues.

### Effects of tryptophan and arginine on the growth and development of *P. palustre*

Tryptophan is a precursor for the synthesis of various hormones in plants, which not only regulates plant growth and development, tissue regeneration, inflorescence opening, and other processes through the synthesis of signaling molecules such as IAA and melatonin (Zhao, 2012; Back et al., 2016; Naureen et al., 2022), but also participates in plant defense responses (Sun et al., 2015; Li B. et al., 2019). In this study, when comparing the metabolic patterns of stems and leaves, we found that the differential abundance of the tryptophan metabolism pathway showed significant up-regulation in both stems and leaves with the growth of *P. palustre*. Compared with the S1 stage, the accumulation abundance of tryptophan increased in the S2 and S3 stages. Therefore, it was speculated that tryptophan might play an important role in the growth and development of *P. palustre*. Further understanding the mechanism that tryptophan participated in the growth and development of *P. palustre* would be of great significance for further elucidating the metabolic regularity of *P. palustre*.

Arginine is an important nitrogen source for plants and a precursor for the synthesis of signaling molecules *in vivo* (Winter et al., 2015; Wang et al., 2021a). Arginine played an important role in the senescence process of *Poplar* and an accumulation of arginine in stems was observed during senescence (Couturier et al., 2010). In this study, the results showed that the differential abundance of the arginine biosynthesis pathway was significantly up-regulated in stem tissues with the growth of *P. palustre* (**Figure 7**). It was consistent with the findings of Couturier et al. (2010). In addition, arginine enhances the synthesis of soluble sugars, flavonoids, proline, free amino acids, and phenolics in *Triticum aestivuml* under normal or stressful conditions (Hussein et al., 2022). Our results showed that monosaccharides and flavonoids were significantly enriched during the S2 stage. It was indicated that arginine might also have the same ability to promote the synthesis of sugars and flavonoids in the growth and development of *P. palustre*. Thus, arginine might have potential research and application value in improving the quality of *P. palustre*.

### Screening of potential markers based on widely targeted metabolomics

Due to the complexity of plant chemical components, the diversity of different origins and harvesting seasons, and the mixture of non-medicinal components, the current quality standards for traditional Chinese medicine still face challenges in evaluating the overall chemical consistency of traditional Chinese medicine. Screening potential markers and using them as quality control indicators is of great significance for ensuring the effectiveness and safety of drugs (Zeng et al., 2023). In addition, using metabolites themselves as markers can often more intuitively reflect the differences between samples. Combining plant metabolomics with network pharmacology can also serve as an effective and comprehensive method for discovering potential active ingredients in traditional Chinese medicine (Liang et al., 2021; Li et al., 2021b). In this study, the screening of potential metabolic markers of *P. palustre* not only helped to reveal its metabolic characteristics at different growth stages and among different tissues but also laid the foundation for the search for secondary metabolic compounds with potential utilization value as well as the further development and utilization of *P. palustre*.

## Conclusions

In this study, we performed widely targeted metabolomics on stems and leaves of three stages of *P. palustre* based on the UPLC-MS/MS detection platform, and a total of 1228 metabolites were detected, including 241 phenolic acids, 203 flavonoids, 152 lipids, 128 terpenes, 66 alkaloids, 79 organic acids, 44 lignans, 9 quinones, 106 amino acids, 68 nucleotide derivatives, 74 saccharides, 1 tannin and so on. Additionally, we identified 13, 10, and 23 potential markers in leaf, stem, and leaf vs. stem comparison groups. Generally, the important monosaccharides and flavonoids that composed the *P. palustre* gum were enriched in the leaf tissues during the S2 stages, suggesting that harvesting *P. palustre* during this stage might maximize its yield and quality. Furthermore, significant variations in the expression of metabolic pathways such as tryptophan metabolism, galactose metabolism, and arginine biosynthesis contribute to the metabolic differences observed in the leaves. The current study not only revealed the metabolic changes during the growth and development of *P. palustre* and provided theoretical support for its cultivation and breeding but also laid the foundation for future research on the key medicinal components of *P. palustre* and its pharmacology and pharmacodynamics.

## Data availability statement

The original contributions presented in the study are included in the article/**Supplementary Material**. Further inquiries can be directed to the corresponding author/s.

## Author contributions

SH and ZC: Writing – original draft, Investigation, and Methodology. HC: Writing – original draft and Formal analysis. CQ and MX: Writing – original draft. and Data curation. FW: Conceptualization, Supervision, and Writing – review & editing. DT: Conceptualization, Funding acquisition, and Writing – review & editing.
