# Supplementary material for: Widely targeted metabolomics reveals the phytoconstituent changes in Platostoma palustre leaves and stems at different growth stages
Source: Front Plant Sci. 2024 Jun 18;15:1378881. doi: 10.3389/fpls.2024.1378881 (PMC11217517; doi:10.3389/fpls.2024.1378881)
Supplement: Supplementary file 1 [file Table_1.docx]

Supplementary table 1 Table of metabolite detection in stems and leaves of *P. palustre* at different growth stages

| Meta Class | All-meta | SS1 | SS2 | SS3 | LS1 | LS2 | LS3 |
| --- | --- | --- | --- | --- | --- | --- | --- |
| Phenolic acids | 241 | 239 | 240 | 239 | 240 | 237 | 238 |
| Flavonoids | 203 | 200 | 200 | 195 | 203 | 202 | 203 |
| Organic acids | 79 | 78 | 78 | 79 | 77 | 79 | 79 |
| Terpenoids | 128 | 128 | 128 | 128 | 128 | 127 | 128 |
| Alkaloids | 66 | 65 | 64 | 65 | 66 | 65 | 66 |
| Amino acids and derivatives | 106 | 105 | 106 | 106 | 106 | 106 | 106 |
| Nucleotides and derivatives | 68 | 68 | 68 | 68 | 68 | 68 | 68 |
| Lipids | 152 | 152 | 151 | 151 | 152 | 152 | 152 |
| Lignans and Coumarins | 44 | 44 | 44 | 44 | 43 | 44 | 44 |
| Quinones | 9 | 9 | 9 | 9 | 9 | 9 | 9 |
| Tannins | 1 | 1 | 1 | 1 | 1 | 1 | 1 |
| Others | 131 | 131 | 131 | 131 | 131 | 130 | 131 |
| All numbers | 1228 | 1220 | 1220 | 1216 | 1224 | 1220 | 1225 |

Note: Meta Class, metabolite class; All-meta, the total number of metabolites of the class detected in *P. palustre*; SS1, SS2, SS3, and LS1, LS2, LS3, the number of metabolites detected in the first, second, and third phases of stems and leaves, respectively.

| Supplementary table 2 Differential metabolites in the stems and leaves of *P. palustre* at different growth stages | | | | | | | | | | | | | | | | |
| --- | --- | --- | --- | --- | --- | --- | --- | --- | --- | --- | --- | --- | --- | --- | --- | --- |
| **LS1_VS_LS2** | Metabolites | number | up**↑** | down**↓** |  | **S1_VS_SS2** | Metabolites | number | up**↑** | down**↓** |  | **SS1_VS_LS1** | Metabolites | number | up**↑** | down**↓** |
|  | Alkaloids | 25 | 22 | 3 |  |  | Alkaloids | 30 | 25 | 5 |  |  | Alkaloids | 20 | 10 | 10 |
|  | Flavonoids | 40 | 26 | 14 |  |  | Flavonoids | 78 | 49 | 29 |  |  | Flavonoids | 152 | 130 | 22 |
|  | Lipids | 38 | 15 | 23 |  |  | Lipids | 39 | 4 | 35 |  |  | Lipids | 66 | 18 | 48 |
|  | Organic acids | 32 | 8 | 24 |  |  | Organic acids | 18 | 12 | 6 |  |  | Organic acids | 32 | 23 | 9 |
|  | Phenlic acids | 70 | 51 | 19 |  |  | Phenolic acids | 70 | 38 | 32 |  |  | Phenolic acids | 149 | 67 | 82 |
|  | Quinones | 5 | 1 | 4 |  |  | Quinones | 5 | 2 | 3 |  |  | Quinones | 4 | 2 | 2 |
|  | Terpenoids | 82 | 71 | 11 |  |  | Terpenoids | 40 | 33 | 7 |  |  | Terpenoids | 62 | 31 | 31 |
|  | Saccharides | 13 | 1 | 12 |  |  | Saccharides | 18 | 15 | 3 |  |  | Saccharides | 31 | 28 | 3 |
|  | Vitamin | 9 | 2 | 7 |  |  | Vitamin | 6 | 3 | 3 |  |  | Vitamin | 14 | 13 | 1 |
|  | Amino acids and derivatives | 47 | 21 | 26 |  |  | Amino acids and derivatives | 33 | 22 | 11 |  |  | Amino acids and derivatives | 37 | 22 | 15 |
|  | Lignans and Coumarins | 15 | 11 | 4 |  |  | Lignans and Coumarins | 16 | 11 | 5 |  |  | Lignans and Coumarins | 34 | 24 | 10 |
|  | Nucleotides and derivatives | 29 | 6 | 23 |  |  | Nucleotides and derivatives | 33 | 12 | 21 |  |  | Nucleotides and derivatives | 35 | 19 | 16 |
|  | Others | 13 | 11 | 2 |  |  | Others | 10 | 5 | 5 |  |  | Others | 22 | 12 | 10 |
|  | **All sig diff** | 418 | 246 | 172 |  |  | **All sig diff** | 396 | 231 | 165 |  |  | **All sig diff** | 658 | 399 | 259 |
| **LS2_VS_LS3** | Alkaloids | 17 | 7 | 10 |  | **SS2_VS_SS3** | Alkaloids | 32 | 23 | 9 |  | **SS2_VS_LS2** | Alkaloids | 38 | 23 | 15 |
|  | Flavonoids | 17 | 12 | 5 |  |  | Flavonoids | 30 | 7 | 23 |  |  | Flavonoids | 147 | 121 | 26 |
|  | Lipids | 41 | 39 | 2 |  |  | Lipids | 19 | 8 | 11 |  |  | Lipids | 53 | 24 | 29 |
|  | Organic acids | 8 | 1 | 7 |  |  | Organic acids | 15 | 6 | 9 |  |  | Organic acids | 27 | 11 | 16 |
|  | Phenolic acids | 35 | 14 | 21 |  |  | Phenolic acids | 45 | 14 | 31 |  |  | Phenolic acids | 122 | 69 | 53 |
|  | Quinones | 5 | 5 | 0 |  |  | Quinones | 5 | 5 | 0 |  |  | Quinones | 8 | 3 | 5 |
|  | Terpenoids | 38 | 35 | 3 |  |  | Terpenoids | 36 | 34 | 2 |  |  | Terpenoids | 67 | 45 | 22 |
|  | Saccharides | 27 | 3 | 24 |  |  | Saccharides | 13 | 1 | 12 |  |  | Saccharides | 22 | 10 | 12 |
|  | Vitamin | 2 | 0 | 2 |  |  | Vitamin | 2 | 0 | 2 |  |  | Vitamin | 11 | 8 | 3 |
|  | Amino acids and derivatives | 34 | 11 | 23 |  |  | Amino acids and derivatives | 32 | 22 | 10 |  |  | Amino acids and derivatives | 41 | 17 | 24 |
|  | Lignans and Coumarins | 7 | 3 | 4 |  |  | Lignans and Coumarins | 14 | 2 | 12 |  |  | Lignans and Coumarins | 28 | 20 | 8 |
|  | Nucleotides and derivatives | 33 | 24 | 9 |  |  | Nucleotides and derivatives | 12 | 5 | 7 |  |  | Nucleotides and derivatives | 22 | 7 | 15 |
|  | Others | 10 | 5 | 5 |  |  | Others | 8 | 2 | 6 |  |  | Others | 26 | 20 | 6 |
|  | **All sig diff** | 274 | 159 | 115 |  |  | **All sig diff** | 263 | 129 | 134 |  |  | **All sig diff** | 612 | 378 | 234 |
| **LS1_VS_LS3** | Alkaloids | 27 | 23 | 4 |  | **SS1_VS_SS3** | Alkaloids | 34 | 29 | 5 |  | **SS3_VS_LS3** | Alkaloids | 27 | 11 | 16 |
|  | Flavonoids | 44 | 27 | 17 |  |  | Flavonoids | 79 | 32 | 47 |  |  | Flavonoids | 155 | 131 | 24 |
|  | Lipids | 55 | 45 | 10 |  |  | Lipids | 47 | 11 | 36 |  |  | Lipids | 57 | 40 | 17 |
|  | Organic acids | 32 | 7 | 25 |  |  | Organic acids | 39 | 19 | 20 |  |  | Organic acids | 30 | 14 | 16 |
|  | Phenolic acids | 55 | 41 | 14 |  |  | Phenolic acids | 89 | 41 | 48 |  |  | Phenolic acids | 130 | 83 | 47 |
|  | Quinones | 4 | 1 | 3 |  |  | Quinones | 2 | 2 | 0 |  |  | Quinones | 5 | 2 | 3 |
|  | Terpenoids | 88 | 83 | 5 |  |  | Terpenoids | 63 | 56 | 7 |  |  | Terpenoids | 60 | 41 | 19 |
|  | Saccharides | 36 | 3 | 33 |  |  | Saccharides | 17 | 10 | 7 |  |  | Saccharides | 33 | 15 | 18 |
|  | Vitamin | 7 | 2 | 5 |  |  | Vitamin | 6 | 3 | 3 |  |  | Vitamin | 13 | 8 | 5 |
|  | Amino acids and derivatives | 43 | 11 | 32 |  |  | Amino acids and derivatives | 43 | 26 | 17 |  |  | Amino acids and derivatives | 42 | 10 | 32 |
|  | Lignans and Coumarins | 12 | 6 | 6 |  |  | Lignans and Coumarins | 15 | 6 | 9 |  |  | Lignans and Coumarins | 32 | 25 | 7 |
|  | Nucleotides and derivatives | 26 | 10 | 16 |  |  | Nucleotides and derivatives | 39 | 13 | 26 |  |  | Nucleotides and derivatives | 34 | 20 | 14 |
|  | Others | 12 | 9 | 3 |  |  | Others | 9 | 4 | 5 |  |  | Others | 25 | 20 | 5 |
|  | **All sig diff** | 441 | 268 | 173 |  |  | **All sig diff** | 482 | 252 | 230 |  |  | **All sig diff** | 643 | 420 | 223 |

Supplementary table 3 Potential biomarkers in stems and leaves of *P. palustre* at different growth stages

| **LS1_VS_LS2** | | | | |  | **LS2_VS_LS3** | | | | |  | **LS1_VS_LS3** | | | | |
| --- | --- | --- | --- | --- | --- | --- | --- | --- | --- | --- | --- | --- | --- | --- | --- | --- |
| Compounds | Level | VIP | P-value | Log2FC |  | Compounds | Level | VIP | P-value | Log2FC |  | Compounds | Level | VIP | P-value | Log2FC |
| 2,3,19,23-Tetrahydroxyurs-12-en-28-oic acid | 1 | 1.17E+00 | 4.75E-04 | -16.46 |  | 2,3,19,23-Tetrahydroxyurs-12-en-28-oic acid | 1 | 1.23E+00 | 2.59E-03 | 16.74 |  | 2-Isopropylmalic Acid | 1 | 1.17E+00 | 7.13E-04 | -4.69 |
| 2-Oxoheptanedionic acid | 3 | 1.17E+00 | 3.33E-03 | 14.42 |  | Benzyl-(2''-O-glucosyl)glucoside* | 3 | 1.23E+00 | 1.30E-02 | 12.88 |  | 2-Oxoheptanedionic acid | 3 | 1.17E+00 | 5.23E-04 | 14.73 |
| Isovanillic Acid | 2 | 1.17E+00 | 9.35E-03 | -13.61 |  | Furanofructosyl-α-D-(6-mustard acyl)glucoside | 2 | 1.23E+00 | 2.79E-02 | -11.51 |  | Creatine | 3 | 1.17E+00 | 6.77E-04 | 13.47 |
| Anthranilic Acid | 3 | 1.17E+00 | 1.92E-03 | -14.14 |  | Isochlorogenic acid B | 3 | 1.23E+00 | 8.03E-03 | 12.97 |  | Demethyl coniferin | 2 | 1.17E+00 | 3.18E-03 | -12.32 |
| Creatine | 3 | 1.17E+00 | 6.35E-03 | 13.70 |  | Isochlorogenic acid C | 2 | 1.23E+00 | 3.39E-03 | -15.60 |  |  |  |  |  |  |
| D-Arabinose | 2 | 1.17E+00 | 1.40E-03 | -20.71 |  | N-Alpha-Acetyl-L-Asparagine | 2 | 1.23E+00 | 3.06E-05 | -5.51 |  |  |  |  |  |  |
| Isochlorogenic acid C | 2 | 1.17E+00 | 3.39E-03 | 15.60 |  |  |  |  |  |  |  |  |  |  |  |  |
| **SS1_VS_SS2** | | | | |  | **SS2_VS_SS3** | | | | |  | **SS1_VS_SS3** | | | | |
| Compounds | Level | VIP | P-value | Log2FC |  | Compounds | Level | VIP | P-value | Log2FC |  | Compounds | Level | VIP | P-value | Log2FC |
| 1,5,7-trihydroxy-6-methoxy-2-methoxymethylanthraquinone | 2 | 1.21E+00 | 5.07E-04 | 5.57 |  | 4-Acetamidobutyric acid | 3 | 1.29E+00 | 1.26E-05 | -4.46 |  | 1,5,7-trihydroxy-6-methoxy-2-methoxymethylanthraquinone | 2 | 1.17E+00 | 3.62E-04 | 9.01 |
| Dicaffeoylspermine | 2 | 1.20E+00 | 3.02E-02 | -12.72 |  | nepetoidin B | 1 | 1.29E+00 | 2.88E-04 | 3.74 |  | Furanofructosyl-α-D-(6-mustard acyl)glucoside | 2 | 1.17E+00 | 6.65E-03 | 11.15 |
| Furanofructosyl-α-D-(6-mustard acyl)glucoside | 2 | 1.21E+00 | 6.56E-04 | 10.78 |  | Isochlorogenic acid C | 2 | 1.29E+00 | 1.48E-03 | -15.43 |  | Hesperetin-6-C-glucoside-7-O-glucoside | 3 | 1.17E+00 | 3.27E-03 | -13.41 |
| Hesperetin-6-C-glucoside-7-O-glucoside | 3 | 1.21E+00 | 3.27E-03 | -13.41 |  |  |  |  |  |  |  | Isochlorogenic acid C | 2 | 1.17E+00 | 1.78E-03 | -15.32 |
| Luteolin-7,3'-di-O-glucoside* | 2 | 1.20E+00 | 1.64E-02 | -13.55 |  |  |  |  |  |  |  | LysoPG 16:1 | 3 | 1.17E+00 | 2.82E-03 | -8.31 |
| LysoPG 16:1 | 3 | 1.21E+00 | 2.82E-03 | -8.31 |  |  |  |  |  |  |  |  |  |  |  |  |
| **SS1_VS_LS1** | | | | |  | **SS2_VS_LS2** | | | | |  | **SS3_VS_LS3** | | | | |
| Compounds | Level | VIP | P-value | Log2FC |  | Compounds | Level | VIP | P-value | Log2FC |  | Compounds | Level | VIP | P-value | Log2FC |
| 2-O-Salicyl-6-O-Galloyl-D-Glucose | 1 | 1.12E+00 | 2.40E-03 | 13.37 |  | 2,3,19,23-Tetrahydroxyurs-12-en-28-oic acid | 1 | 1.12E+00 | 3.23E-04 | -14.55 |  | 2-O-Salicyl-6-O-Galloyl-D-Glucose | 1 | 1.12E+00 | 2.47E-03 | 14.30 |
| Argininosuccinic acid | 3 | 1.12E+00 | 3.80E-03 | 12.48 |  | 2-O-Salicyl-6-O-Galloyl-D-Glucose | 1 | 1.12E+00 | 4.17E-03 | 14.01 |  | Demethyl coniferin | 2 | 1.12E+00 | 5.14E-04 | -14.02 |
| Creatine | 3 | 1.12E+00 | 6.50E-03 | -12.53 |  | Isovanillic Acid | 2 | 1.12E+00 | 1.29E-03 | -17.23 |  | Eriodictyol-7-O-(6''-O-p-coumaroyl)glucoside | 2 | 1.12E+00 | 1.08E-03 | 14.44 |
| Eriodictyol-7-O-(6''-O-p-coumaroyl)glucoside | 2 | 1.12E+00 | 4.61E-05 | 15.18 |  | Anthranilic Acid | 3 | 1.12E+00 | 2.76E-04 | -14.05 |  | Hesperetin-6-C-glucoside-7-O-glucoside | 3 | 1.12E+00 | 1.88E-03 | 17.69 |
| Farrerol-5,7-di-O-glucoside | 3 | 1.12E+00 | 8.59E-03 | 13.23 |  | Argininosuccinic acid | 3 | 1.12E+00 | 2.39E-03 | 11.30 |  | Kaempferol-3-O-glucorhamnoside* | 1 | 1.12E+00 | 7.39E-03 | 19.69 |
| Fraxetin-7,8-di-O-glucoside | 2 | 1.12E+00 | 7.33E-05 | -13.02 |  | Isochlorogenic acid B | 3 | 1.12E+00 | 6.50E-05 | -12.65 |  | Kaempferol-3-O-neohesperidoside* | 1 | 1.12E+00 | 2.58E-03 | 19.68 |
| Gallocatechin-(4α→8)-catechin | 3 | 1.12E+00 | 6.19E-03 | 12.88 |  | Robinin | 2 | 1.12E+00 | 6.92E-05 | 15.24 |  | Kaempferol-3-O-robinoside-7-O-rhamnoside (Robinin) | 2 | 1.12E+00 | 5.83E-04 | 14.90 |
| Isochlorogenic acid C | 2 | 1.12E+00 | 1.78E-03 | -15.32 |  | Lithospermoside | 2 | 1.12E+00 | 4.71E-04 | 13.02 |  | Lithospermoside | 2 | 1.12E+00 | 3.40E-03 | 13.37 |
| Tiliroside | 1 | 1.12E+00 | 4.80E-04 | 8.95 |  | Luteolin-7,3'-di-O-glucoside* | 2 | 1.12E+00 | 6.63E-03 | 19.85 |  | Luteolin-7,3'-di-O-glucoside* | 2 | 1.12E+00 | 8.13E-03 | 19.45 |
| Robinin | 2 | 1.12E+00 | 2.85E-03 | 14.97 |  |  |  |  |  |  |  | Luteolin-7-O-(2''-O-rhamnosyl)rutinoside | 2 | 1.12E+00 | 4.61E-03 | 14.82 |
| Lithospermoside | 2 | 1.12E+00 | 6.68E-03 | 12.52 |  |  |  |  |  |  |  | Luteolin-7-O-neohesperidoside (Lonicerin) | 3 | 1.12E+00 | 7.39E-03 | 19.69 |

Level: Metabolite identification level (Level 1: The matching score between the secondary mass spectrometry and RT of the sample substance and the database substance is above 0.7; Level 2: The matching score between the secondary mass spectrometry and RT of the sample substance and the database substance is 0.5-0.7; Level 3: The sample substances Q1, Q3, RT, DP, CE are consistent with the database substances).
